# Light-induced epigenetic modifications in the hypothalamus during avian embryonic development enhance phenotypic plasticity

**DOI:** 10.3389/fcell.2025.1573705

**Published:** 2025-06-26

**Authors:** Joanna Bartman, Tali Rosenberg, Hadar Parnas, Ronit Yosofov, Natalie Avital-Cohen, Ron Weiss, Gal Harmatz, Israel Rozenboim, Asaf Marco

**Affiliations:** The Department of Animal Science, The Robert H. Smith Faculty of Agriculture, Food and Environment, The Hebrew University of Jerusalem, Rehovot, Israel

**Keywords:** green monochromatic illumination, epigenetic modifications, embryonic development, hypothalamus, avian

## Abstract

**Introduction:**

Early brain development is highly sensitive to environmental cues, which can exert lasting phenotypic effects. Targeted external interventions during this critical period can shape developmental trajectories and influence an animal’s phenotype. Aligned with this concept, fetal responses to light-induced stimuli—varying in wavelength, frequency, and duration—are thought to facilitate adaptive reactions that enhance phenotypic plasticity and equip organisms to meet environmental challenges.

**Methods:**

In this study, broiler eggs were exposed to green monochromatic illumination (GMI) either continuously throughout incubation (Green) or during the final 3 days only (G3D) and compared to dark and white light controls.

**Results:**

Genome-wide analyses revealed significant transcriptional changes in the hypothalamus of the G3D group, identifying over 500 differentially expressed genes related to growth, metabolism, appetite, and immunity. Epigenetically, GMI exposure increased phosphorylated CREB1 (pCREB1) binding levels and chromatin accessibility at specific gene promoters, underscoring the role of light-induced developmental programming. Notably, these effects were exclusive to the G3D group, highlighting the last 3 days of incubation as a critical window for intervention. In G3D, cFOS immunostaining revealed heightened hypothalamic responsiveness to a post-hatch green light pulse, indicating that in-ovo GMI primed the brain’s circuitry for future stimuli. Mechanistically, our findings suggest that GMI-induced hypothalamic adaptations are mediated, at least partially, through retinal green photoreceptors. Pre-exposure to blue light, which disrupts green photoreceptor activity, reduced retinal green opsin levels and nullified the epigenetic changes typically observed in the G3D group. Last, G3D chicks exhibited enhanced growth and improved food conversion ratios (FCR), particularly during early post-hatch development. Consistent with our transcriptomic and epigenetic data, the BG6D group showed no significant changes in body weight or FCR.

**Discussion:**

Collectively, these findings highlight how specific wavelengths and precise timing of light exposure during embryogenesis can shape post-hatch phenotypes through transcriptional and epigenetic mechanisms.

## 1 Introduction

In the early stages of development, the brain demonstrates an extraordinary susceptibility to environmental stimuli and modifications. These stimuli have the capacity to elicit long-term effects, shaping the phenotype of the organism later in life (Feil and Fraga, 2012). Largely owing to the avian embryo’s development outside the mother’s body, early environmental manipulation allows for the isolation of maternal effects and provides an effective means to modulate and guide offspring developmental trajectories with minimal confounding variables (Carvalho et al., 2023; Carvalho et al., 2020; Porter, 2000; Kisliouk et al., 2024). Previous studies (Porter, 2000; Kisliouk et al., 2024); have demonstrated that in-ovo manipulations can exert lasting effects on offspring, offering a straightforward and practical approach to enhance growth performance and increase resiliency.

Among different environmental stimuli, light exposure at varying wavelengths, frequencies, and durations was demonstrated to impact physiological, cellular, and molecular aspects. Though not fully understood, it is suggested that fetal responses to light-induced stimuli facilitate an adaptive (or maladaptive) reaction that promotes phenotypic plasticity and prepares organisms to cope with environmental challenges (Villamizar et al., 2014). These responses encompass changes in the control mechanisms governing circadian rhythms, metabolism, fertility, and growth (Piestun et al., 2015; Yalcin et al., 2022; Lucon-Xiccato et al., 2023; Septriani et al., 2021). While the mechanism by which light triggers changes in the brain remains to be fully elucidated, several studies have proposed a connection between the activation of retinal photoreceptors, the visual cortex, and subsequent responses in different brain regions (Fernandez et al., 2018; Nash et al., 2019). Furthermore, research has revealed that various vertebrates possess extra-retinal photoreceptors (ERPRs), located in other tissues such as the skin, pineal gland, and hypothalamus (Soni et al., 2021; Yoshikawa and Oishi, 1998; Pérez et al., 2019). These ERPRs appear to play crucial roles in diverse physiological responses, including the detection of seasonal changes and regulation of fertility (García-Fernández et al., 2015).

Accordingly, our previous studies along with those of others, have shown that artificial targeted illumination at red wavelengths significantly affects the reproductive system in birds (Oishi and Lauber, 1973; Mobarkey et al., 2010), whereas shorter wavelengths, such as green, mainly inhibit it (Benoit and Assenmacher, 1966; Mobarkey et al., 2013; Bédécarrats, 2015). Moreover, studies across different species have demonstrated that prolonged exposure to blue wavelengths can bleach and disrupt the activity of green photoreceptors, subsequently interfering with the signals transmitted from the retina to the brain (Grimm et al., 2001; Grimm et al., 2000). Thus, adjusting the illumination or intensifying a specific wavelength can lead to the development of distinct phenotypes. Further supporting evidence has shown that prolonged exposure to green monochromatic illumination (GMI) during avian incubation (in-ovo) significantly increases post-hatch body and muscle weight (Dishon et al., 2021). The observed phenotype remained stable and consistent throughout adulthood, suggesting long-term phenotypic programming as a result of in-ovo GMI exposure. Moreover, these investigations have highlighted the temporal sensitivity of these mechanisms, identifying a critical time window during the last 3 days before hatching where the most significant impact was observed. Particularly, phenotypic effects induced by both acute and chronic GMI exposures are partly mediated by transcriptional changes in the hypothalamic-pituitary–somatotropic axis, which includes the secretion of growth hormone-releasing hormone (GHRH) and the subsequent stimulation of growth hormone (GH) and insulin-like growth factor 1 (IGF-1) (Dishon et al., 2021). Beyond its role in growth regulation, the hypothalamus is a vital brain region that governs various physiological processes, including appetite, immunity, and metabolic pathways (Suzuki et al., 2012; Belluardo et al., 1987; Kim et al., 2020). Hence, it is plausible to consider that exposure to GMI at different developmental stages could elicit alterations across a diverse range of biological pathways.

Henceforth, in this study, we employed broiler embryos to investigate whether acute and prolonged exposure to GMI during the critical developmental periods, could epigenetically reprogram the ‘set points’ of hypothalamic circuits. To address these questions, broiler eggs were subjected to two distinct lighting regimes: green monochromatic illumination throughout incubation (Green) and green monochromatic illumination only during the last 3 days of incubation (G3D). These animals were compared to two control groups (dark and white polychromatic illumination). RNA-seq analysis of hypothalamic samples at day of hatch (DOH) revealed minimal gene expression changes in the White and Green groups compared to control. In contrast, the G3D group showed significant transcriptional alterations with over 500 differentially expressed genes (DEGs) related to growth, appetite, and metabolism. Correspondingly, this group also exhibited increased phosphorylated CREB1 (pCREB1) and Histone H3 Lysine 27 acetylation (H3K27ac) binding levels at gene promoters. Next, we investigated whether hypothalamic responsiveness to GMI was enhanced following epigenetic reprogramming. To achieve that, broiler eggs were incubated under four conditions (Control, White, Green, G3D). At hatch, we subjected chicks to a post-hatch green light pulse and assessed cFOS levels, a broad marker of neuronal activity. While no significant differences were observed in the Green or Control groups, the G3D group showed a significant increase in cFOS expression, indicating heightened post-hatch hypothalamic sensitivity to green light.

In a separate set of experiments, pre-exposure to blue light before GMI significantly reduced retinal green opsin levels and completely nullified the epigenetic changes observed in the G3D group. Consistent with the molecular effects seen at DOH, the G3D group displayed a mild increase in post-hatch body weight (from DOH to day 16) and improved food conversion ratio (FCR), reflecting enhanced metabolic efficiency. Collectively, these findings underscore the pivotal role of specific wavelengths and timing of light exposure in shaping broilers’ developmental trajectories via transcriptional and epigenetic mechanisms. Furthermore, our results reveal that GMI exerts broad effects on hypothalamic function, enhancing growth and metabolic efficiency while preserving animal health and wellbeing.

## 2 Methods

### 2.1 Animals and tissue processing

All trials and procedures were approved by the Animal Care and Welfare Committee of The Hebrew University of Jerusalem, Israel on the seventh of August 2020, Research number AG- 20–1631-3, and on the first of February 2024, Research number AG- 24- 17,467–4.

Three hundred fertile broiler eggs (Ross 308), with an average weight of 60.5 ± 0.7 g, were incubated during three individual trials in different lighting treatments: dark (control), white for all incubation and hatching periods (White), GMI for the whole duration of incubation and hatching (Green), GMI only in the last 3 days of incubation - hatching period (G3D) and blue from embryonic day 16 (E16) until embryonic day 18 (E18) followed by GMI on last 3 days of incubation (BG6D). All illumination systems were located above the egg trays, separated with light-proof dividers, and set to an even illumination intensity of 0.1 W/m2 (LI-CORE light meter, Lincoln, USA). Monochromatic and polychromatic wavelengths were measured using UPRtek MK3505 Handheld Spectrometer® for spectral analysis and detection of spectral bleeding. On day of hatch (DOH), Hypothalamic tissues were collected from all four groups and divided into two hemispheres; one for gene expression analysis via RNA sequencing, and the second hemisphere was used to determine alteration in chromatin accessibility in the vicinity of targeted genes. All samples were snap-frozen in liquid nitrogen immediately and stored at −80°C.

12 chicks (3 chicks from control, White, Green, and G3D treatment groups) at DOH were subjected (post-hatch) to 5 min of green monochromatic pulse of light, followed by 30 min in darkness. Overall, three chicks from all treatment groups (with and without pulse) were sacrificed and whole brains were collected for immunofluorescence staining. Brains were drop-fixated in 4% Paraformaldehyde and stored in PBS azide at 4°C until slicing.

At the last trial, 96 chicks were incubated in Control, Green, G3D, and BG6D illumination regimes. At DOH, the chicks were transferred to a rearing facility where they were housed in three identical rooms divided into four equal pens (1.5 m^2^). In each section, we reared chicks from each treatment group. Animals were reared according to primary breeder recommendations according to the ROSS broiler management handbook and illuminated with standard white polychromatic lighting during the entire duration of the experiment.

Weekly body weights (BW) were recorded. Between day 8 and the end of the experiment, at 35 days of age, feed consumption was recorded every other day (total of 12 sections) for FCR assessment. FCR was measured for each animal after each weighting by the following formula:

$$\left\{\frac{\left(\frac{\text{Feed}\text{cosnsumed}\text{in}\text{eac}h\text{section}}{\text{Number}\text{of}\text{animals}\text{in}\text{each}\text{section}}\right)}{\text{Weight}\text{gain}\text{of}\text{each}\text{animal}}\right\}$$

### 2.2 RNA isolation and sequencing

RNA was extracted from one of the hypothalamic hemispheres. Total RNA was isolated using TriReagent (Molecular Research Center, Cincinnati, OH) according to the manufacturer’s instructions. RNA quantity and purity were measured using TapeStation (Agilent Technologies, Santa Clara, CA). RNA-seq was conducted at the Crown GenomicsInstitute of the Nancy and Stephen Grand Israel National Center for Personalized Medicine (INCPM), Weizmann Institute of Science (Rehovot, Israel). As previously described by Rosenberg et al. (2022), an adaptation of the bulk MARS-Seq protocol (Keren-Shaul et al., 2019) was used to generate RNA-seq libraries for expression profiling of all groups (Control (dark), White, Green, G3D; n = 3–4/group). Briefly, 200 ng of input RNA from each sample was barcoded during reverse transcription and pooled. Following Agencourt Ampure XP beads cleanup (Beckman Coulter, Luzerne, Singapore), the pooled samples underwent second-strand synthesis and were linearly amplified by T7 *in vitro* transcription. The resulting RNA was fragmented and converted into a sequencing-ready library by tagging the samples with Illumina sequences during ligation, RT, and PCR. Libraries were quantified by Qubit and TapeStation. Sequencing was performed on a Nextseq 75 cycles high output kit (Illumina).

### 2.3 RNA-seq analysis

Illumina adapters were trimmed from the reads using Cutadapt (V3.4) (Martin, 2011). The resulting reads were mapped onto 3′ UTR regions (1000 bases) of the Gallus genome (Galgal 6.0, UCSD) according to Refseq annotations, using STAR (Dobin et al., 2013) with 82% alignment rate, resulting in an average of ∼12 million mapped reads per sample. Deduplication was carried out by flagging all reads that were mapped to the same gene and had the same UMI. Read counts per gene were calculated using HTseq-count (Anders et al., 2015). Differential gene expression and downstream analyses were performed using DESeq2 (Love et al., 2014) (adjusted p-value <0.05). We assessed the expression of all DEGs, with respect to the Control (dark) group and clustered them with R packages Circlize (0.4.15), ComplexHeatmap (2.18), gridExtra (2.3), viridis (0.6.4). Respective heatmaps were generated by custom R scripts and the Complexheatmap R package. Unique and shared DEGs analysis was performed with InteractiVenn (Heberle et al., 2015). Metascape suite was used for Gene Ontology (GO) analysis (Zhou et al., 2019). The combined score (-log(p) * oddsRatio) of the motif enrichment was calculated by the Enrichr package (Chen et al., 2013). DNase, H3K27ac and H3K4me3 ChIP-seq data were adopted from Pan et al. (2023) to generate gene tracks in IGV (Pan et al., 2023).

### 2.4 Chromatin-immunoprecipitation (ChIP) and qPCR

The remaining hypothalamic hemisphere was cross-linked with 1% formaldehyde (Electron Microscopy Sciences, Hatfield, PA, cat# 15714) for 10 min at room temperature and quenched with glycine quenching solution (Cell Signaling Technology, Danvers, MA, cat# 7005S). Crosslinked samples were resuspended in a ChIP lysis buffer (0.99% SDS, 9.9 mM EDTA, 49.5 mM Tris-HCl pH8.8, protease inhibitor 0.99M), and sonicated (Bioruptor Plus, Diagenode) to release 200–1000 bp fragments, assessed by TapeStation (Agilent Technologies, Santa Clara, CA). Samples were diluted 1:5 with a ChIP dilution buffer (167 mM NaCl, 16.7 mM Tris-HCl pH8, 1.2 mM EDTA, Tritonx1.1, 0.01% SDS). Antibodies (1:50) against pCREB (phospho S133) (Abcam, Cambridge, MA, cat# ab32096, RRID: AB_731734) or H3K27ac (Cell Signaling Technology, Danvers, MA, cat# 8173, RRID: AB_10949503) were immunoprecipitated at 4°C over-night. The samples were then conjugated to Magna ChIP Protein A + G Magnetic Beads (Sigma-Aldrich, St. Louis, MO, cat# 16–663) or Sera Mag SpeedBeads Protein A/G (Cytivia, Marlborough, MA, cat# 1715–2104–010150) at 4°C overnight. Immunocomplexes were washed sequentially with the following buffers: low-salt buffer (0.1% SDS, Tritonx1, 1 mM EDTA, 20 mM Tris-HCl pH8, 0.15M NaCl), high-salt buffer (0.1% SDS, Tritonx1, 2 mM EDTA, 1M Tris-HCl pH8, 0.5M NaCl), and two washes with TE buffer (10 mM Tris-HCl pH8, 1 mM EDTA). The samples were then eluted with the following buffer: (1% SDS, NaHCO3 90 mM), coupled with RNAse A (Cell Signaling Technology, Danvers, MA, cat# 7013) for 20 min at 37°C. Chromatin was deproteinized and de-crosslinked with proteinase K (Thermo Fisher, Waltham, MA, cat# 4333793) and NaCl 175 mM for 4 h at 62°C. Deconjugated samples were heated for 10 min at 95°C. DNA was purified and precipitated using DNA purification buffers and Spin Columns (ChIP, CUT&RUN) 1 Kit (Cell Signaling Technology, Danvers, MA, cat# 14209) or MinElute PCR Purification Kit (QIAGEN, Venlo, Netherlands, cat# 28004). Real-time PCR (qPCR) was performed in CFX Connect Real-Time PCR Detection System (Bio-Rad, Hercules, CA) with PerfeCTa SYBR Green FastMix, ROX (Quanta BioSciences, Gaithersburg, MD, cat# 95072–012). Dissociation curves were analyzed following each real-time PCR to confirm the presence of only one product and the absence of primer dimer formation. Results were normalized to 5% input samples that were not precipitated. The threshold cycle number (Cq) for each tested region in proximity to a specific gene (X) was used to quantify the relative abundance of those binding levels using Formula 2 (Ct ChIP X–Ct input).

The primers used for real-time PCR are listed in the table below (Table 1):

**TABLE 1:** Primer sequences used in ChIP-qPCR to evaluate pCREB and H3K27ac enrichment at promoter regions of targeted genes.

Gene name	Forward sequence	Reverse sequence
Agrp	CTTTGCTGCGTCTCGTCC	CAGCCCCACTCACGTACAA
Gal	TGCACATACCTACACTGGCT	ATGAAGCTCTCCCCAGTG
Ghrh	TGCTCAACCCCGAAAACC	GCACGAGGGAAGGGGTATG
Leprot	CGTACGTCTTAGAGGCACACACA	CTCCCGTCGCCATGGTTA
Pacap	AGCGACGGAATCTTTGCAAA	AATATTGCCCGTCCTCCTCC
Trh	GATGCCCCAAACAAAACAC	TGTCAGGGAGCAGTAAGGC
Vip	GCGTGGCTGCTCATGAATTA	GAGCAATCACGTTACTGGGGA

### 2.5 Immunofluorescence staining

Brains from all eight treatment groups (with and without pulse, n = 3 per group) were embedded in 4% agar solution and coronally sectioned at 40 µm using a vibratome (Leica Biosystems). Two sections per animal were washed in PBST (20 mM Tris, pH 7.4, 150 mM NaCl, 0.05% Tween 20), blocked in PBST +4% BSA (Sigma Aldrich, St. Louis, MO, cat# A3912-100G) for 2 h, and then incubated overnight at 4°C with primary antibody (anti-rabbit cFOS antibody 1:250; Santa Cruz Biotechnology, Dallas, TX, cat# sc-166940, RRID: AB_10609634 or mouse monoclonal CREB1 antibody 1:250, Santa Cruz Biotechnology, Dallas, TX, cat# sc-377154, RRID: AB_3674738, dilution). After washing three times in PBST, the sections were incubated at room temperature for 3 h with a secondary antibody (Anti-Rabbit IgG H&L, Alexa Fluor^®^ 488, Abcam, Cambridge, UK, cat# ab150077, RRID: AB_2630356 or Anti-Mouse Alexa Fluor^®^ 594, Abcam, Cambridge, UK, cat# ab150116, RRID: AB_2650601). Sections were incubated with (4′,6-diamidino-2-phenylindole) DAPI (Sigma-Aldrich, St. Louis, MO, cat# D9542) for 15 min before they were mounted on slides using VECTASHIELD^®^ (Vector Laboratories, Newark,CA, cat# H-1000–10) and imaged using an Andor BC43 confocal microscope (Oxford Instruments, Abingdon, UK). The correct coordinates for the hypothalamus on the coronal slices were determined using a stereotaxic atlas of the chick brain, specifically referencing plates A 7.8, A 8.0, and A 8.2. (Kuenzel and Masson, 1988). Images were processed in Fiji, counting cFOS-positive cells from both hemispheres of the hypothalamus in each image. CREB1 and nuclear DAPI puncta within a defined hypothalamic region of interest (ROI, in mm^2^) were identified using the “Spots” detection model in Imaris 10 microscopy image analysis software (Oxford Instruments). The shortest distance between each CREB1 punctum and the nearest DAPI-labeled nucleus was calculated. A threshold of ≤3 µm was used to define nuclear localization. CREB1 puncta within this distance were classified as nuclear p-CREB1, while those located >3 µm fsrom DAPI were categorized as cytosolic (non-phosphorylated) CREB1.

### 2.6 Western blot

Retinas from the right eye of the hatchlings were homogenized with 2x Leamly sample buffer (120 mM Tris–HCl, pH 6.8, 4% SDS, 20% glycerol, and 10% β-mercaptoethanol). Protein extracts were separated on a 12% SDS-polyacrylamide gel and transferred them to nitrocellulose membranes. The membranes were blocked in Tris-buffered saline with Tween 20 (20 mM Tris, pH 7.4, 150 mM NaCl, and 0.05% Tween 20) + 5% bovine serum albumin (BSA) (Sigma Aldrich, cat# A3912-100G) for 1 h at room temperature and incubated overnight with anti-mouse green opsin (1:1000; Cell Signaling Technology, Danvers, MA, cat# ab150077, RRID: AB_2630356) or anti-rabbit β-actin (1:5000; (Cell Signaling Technology, Danvers, MA, cat# 4970, RRID: AB_2223172) antibodies at 4°C. The membranes were washed and then incubated with HRP conjugated anti-mouse and anti-rabbit secondary antibodies (1:10,000; GE Healthcare) at room temperature for 1h. A chemiluminescent signal was detected using PierceTM ECL Western blotting Substrate (Pierce Biotechnology, Waltham, MS, cat# 32106) by the ChemiDocTM XRS + molecular imager^®^ (BIO-RAD, Hercules, CA, USA), and densitometric analysis was performed using ImageJ open-source software.

### 2.7 Statistical analysis

GraphPad Prism 10.1.2 software was used for all statistical analyses. All data were examined for normality by the Shapiro-Wilk normality test. Sample number (n) included the number of individual animals in each treatment group (indicated in the figure legends). One-way ANOVA followed by Fisher’s LSD multiple comparison tests were used. Figure data are presented as mean ± standard error mean (SEM), with exact n- and p-values for effects, as well as p-values for multiple comparisons reported in the legends.

## 3 Results

### 3.1 In-ovo GMI exposure alters hypothalamic transcriptional programming in pathways related to growth, metabolism, and appetite

**FIGURE 1:**
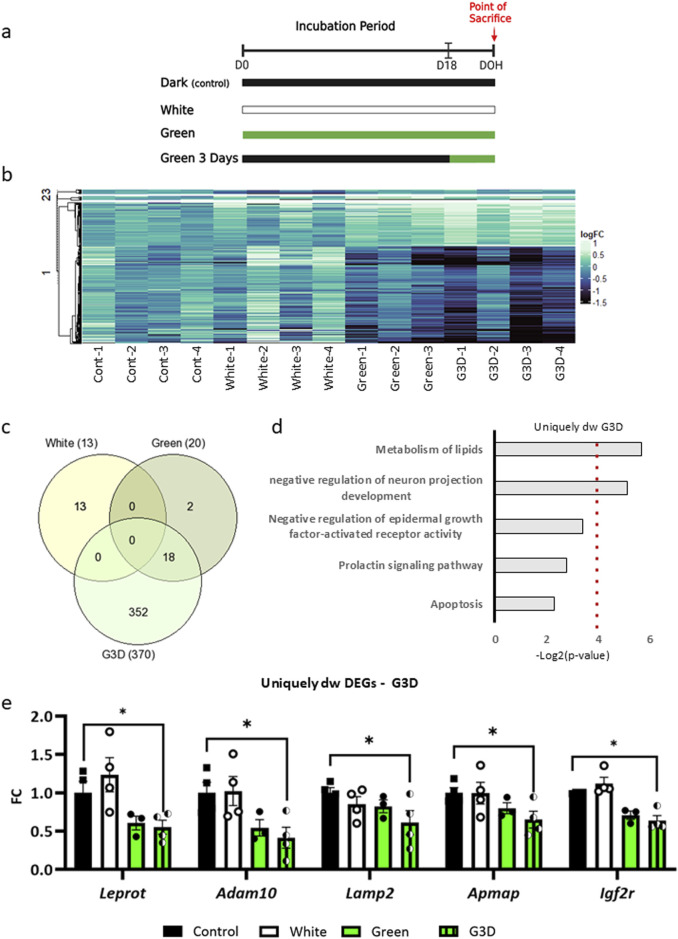
Genome-wide transcriptional profiles in the hypothalamus of male broiler chicks at day of hatch (DOH) induced by in-ovo exposure to various illumination conditions. **(a)**. Illustration of the experimental design; 308 fertile Broiler eggs were exposed to GMI either throughout the entire incubation period (Green) or specifically during the final 3 days before hatching (G3D), with control groups incubated in dark (Control) or white light conditions during 21 days of incubation (White). **(b)**. Heat map displays the clusters of average hypothalamic transcriptional changes (log2FC) of DEGs from the pairwise analysis of White (cluster 1), Green (cluster 2), and G3D (cluster 3) with respect to the Control (dark). **(c)**. Venn diagram shows the overlap between the downregulated DEGs in all three treatment groups compared to control. **(d)**. Gene Ontology (GO) analysis of uniquely downregulated DEGs identified in the G3D group. The x-axis represents -Log2 (p-value). **(e)**. Shown are representative uniquely downregulated DEGs, with normalized counts depicted as Log2FC from the Control group. Data are presented as mean ± SEM. Significant effect between groups is indicated by *p < 0.05, **p < 0.01, ***p < 0.001, ****p < 0.00001, using One-way ANOVA test with LSD’s multiple comparisons.

We have previously shown that in-ovo GMI -exposure significantly increases both body and muscle weight, which were accompanied by a significant increase in hypothalamic GHRH expression (Dishon et al., 2018). Thus, our first objective was to evaluate unbiasedly whether GMI promotes additional hypothalamic transcriptional alterations. Here, avian eggs (Ross 308) were randomly subjected to four distinct lighting regimes during incubation: (i) dark incubation, serving as the control group (Control); (ii) white polychromatic illumination throughout the entire 21-day incubation period (White), aimed at assessing the importance of targeting the green wavelength for the desired effect; (iii) green monochromatic illumination throughout the entire incubation period (Green), aimed at establishing the significance of exposure duration; (iv) and green monochromatic illumination only during the last 3 days of incubation (G3D) (Figure 1a). At DOH, hypothalamic tissues from all aforementioned groups were extracted and subjected to RNA-seq. Principal component analysis (PCA) illustrated the clustering of the Green and G3D groups, showcasing marked differences from the Control and White groups (Supplementary Figure S1). Next, we quantified differentially expressed genes (DEGs) in the three groups (White, Green, G3D), relative to the dark–incubated group (Control), (Supplementary Table S1).

**FIGURE 2:**
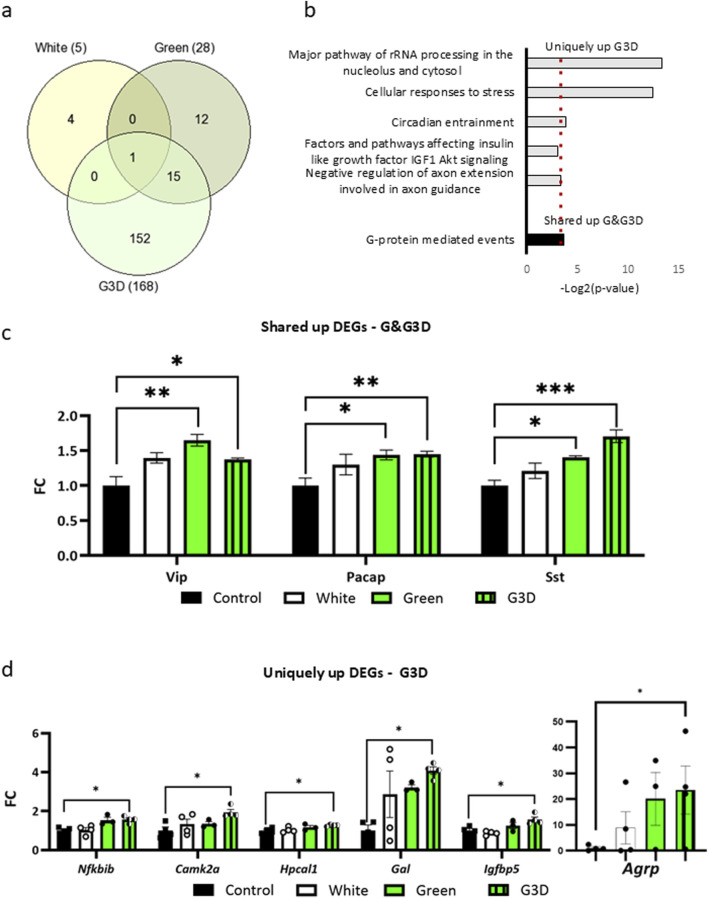
In-ovo exposure to GMI upregulates gene expression in pathways linked to growth, metabolism, and immunity. **(a)**. Venn diagram shows the overlap between the upregulated DEGs in all three treatment groups compared to control. **(b)**. GO analysis of uniquely upregulated DEGs identified in the G3D group (gray bars) and shared DEGs between the Green and G3D groups (black bar). The x-axis represents -Log2 (p-value). **(c)**. Representative upregulated shared DEGs between the Green and G3D groups (Nfkbib, Camk2a, Hpcal1, Galanin, Igfbp5 and Agrp) and **(d)**. Unique DEGs identified in the G3D group (Vip, Pacap, and Somatostatin) (black bar). Normalized counts are depicted as Log2FC from the Control group. Data are presented as mean ± SEM. Significant effect between groups is indicated by *p < 0.05, **p < 0.01, ***p < 0.001, ****p < 0.00001, using One-way ANOVA test with LSD’s multiple comparisons.

These pairwise comparisons uncovered minimal transcriptional changes in the White and Green groups when compared to the control group (13 downregulated genes and five upregulated genes in Control vs. White; 20 downregulated and 28 upregulated DEGs in Control vs. Green, padj <0.05, Figure 1B, heatmap clusters 2 and 3). In contrast, the G3D group exhibited robust significant alterations, with 370 downregulated and 168 upregulated DEGs when compared to the dark–incubated controls (Control vs. G3D, padj <0.05, Figure 1b, heatmap cluster 1). Next, we investigated GMI-induced transcriptomic changes, aiming to identify alterations that were unique to each group as well as those that were shared among them (Figure 1c). While we did not detect any overlaps in the downregulated genes with the White group, we observed 18 DEGs that overlapped between the Green and the G3D groups. Moreover, this analysis revealed 352 DEGs that were uniquely expressed in the G3D group (Figure 1c). Subsequently, we performed Gene Ontology (GO) analysis to pinpoint key biological and molecular pathways within both the shared and uniquely expressed DEGs. While the shared DEGs did not show significant enrichment in any major pathways, the uniquely downregulated genes in the G3D group were predominantly enriched in pathways associated with ‘negative regulation of neuron projection development’, ‘negative regulation of epidermal growth factor-activated receptor activity’, ‘Prolactin signaling pathway’, ‘Apoptosis’ and ‘Metabolism of lipids’ (Figure 1d). Within these pathways we identified the downregulation of *Leprot* [padj: 0.0349] which negatively regulates cell surface expression of leptin receptors (LEPR) and growth hormone receptors (GHR) (Touvier et al., 2009). Thus, it is likely that a GMI-mediated decrease in the expression of this inhibitory agent will lead to an improvement in the hypothalamic responsiveness to leptin and GH signals. Furthermore, we identified several genes, which were previously linked to enhanced insulin sensitivity, decreased inflammation, and oxidative stress (Saftig and Lichtenthaler, 2015); (Kaushik and Cuervo, 2012); (Ma et al., 2016); (Torrente et al., 2020) (ADAM metallopeptidase domain 10 (*Adam10*: [padj = 0.0000415], lysosomal associated membrane protein 2 (*Lamp2*: [padj = 0.0323]), adipocyte plasma membrane–associated protein (*Apmap*: [padj = 0.00345]) and insulin-like growth factor-II receptor (padj = 0.001306], Figure 1e; Supplementary Table S1). Within the upregulated DEGs, 15 genes were found to overlap between the Green and G3D groups (Figure 2a), with a specific enrichment in pathways related to 'G-protein mediated events’ (Figure 2b). This overlap includes genes such as Pituitary adenylate cyclase-activating polypeptide (Pacap) [G3D-padj = 0.0016, Green-padj = 0.02334], Somatostatin (Sst) [G3D-padj = 0.00000, Green-padj = 0.02745], and Vasoactive Intestinal Peptide (Vip) [G3D-padj = 0.004113, Green-padj = 0.006695], which are essential for regulating the HPS axis and play significant roles in controlling appetite and metabolism (Montero et al., 2000); (Guillemin and Gerich, 1976); (Vu et al., 2015) (Figure 2c). These findings suggest that targeted green illumination during embryonic incubation has broad and common effects on growth, feeding behaviors, and metabolic processes.

Uniquely in the G3D group, a distinct set of 152 upregulated DEGs were enriched with pathways such as ‘Cellular responses to stress’, ‘Circadian entrainment’ and ‘Factors and pathways affecting insulin-like growth factor IGF1 -Akt signaling’ (Figure 2b). Within these pathways, noteworthy genes included the master regulator NF-Kappa-B Inhibitor Beta (*Nfkbib*: [padj = 0.04235]), which inhibit Nfkb by complexing with, and trapping it in the cytoplasm (Figure 2d). Previous studies have indicated that Nfkbib suppresses inflammation, thereby aiding in the maintenance of normal hypothalamic function under metabolic challenges, oxidative stress, and inflammation (Huang et al., 2020). Notably, we identified Agouti Related Neuropeptide (*Agrp*: [padj = 0.04579]), Galanin and GMAP Prepropeptide (*Gal*: [padj = 0.01448) and Insulin Like Growth Factor Binding Protein 5 (*Igfbp5*: [padj = 0.00532]), which are potent orexigenic factors (Figure 2d). Previous research has demonstrated that ubiquitous overexpression or central administration of Agrp increases food intake (Denbow and Cline, 2015). Furthermore, these factors synergistically modulate lipid metabolism and insulin sensitivity by activating the AMPK pathway, suggesting that both appetite and metabolic pathways are further affected in the G3D group. Interestingly, the G3D-specific upregulation of Hippocalcin Like 1 (*Hpcal1*), a member of the neuron-specific calcium-binding protein family present in both the retina and brain, was particularly notable among the uniquely upregulated genes. Hpcal1 is involved in the calcium-dependent regulation of rhodopsin phosphorylation and thus may be relevant for light-induced neuronal signaling in the brain. Additionally, uniquely in the G3D group, we observed a significant increase in the transcription of the gene encoding Calcium/Calmodulin-Dependent Protein Kinase II Alpha (*Camk2a*: [padj = 0.02135]) (Figure 2d). CaMK2-mediated CREB phosphorylation plays a crucial role in numerous epigenetic and transcriptional programs within the brain. Consequently, these findings not only corroborate our previous results and observed phenotype (Dis et al., 2021) but also suggest broader-scale hypothalamic transcriptional and molecular alterations induced by GMI, which are dependent on the developmental phase and duration of the manipulation.

### 3.2 GMI-induced modulations in pCREB and H3K27ac are associated with transcriptional alterations

In the next phase, our investigation focused on determining whether the observed alterations in gene expression following the GMI, were mediated through epigenetic modulations. To assess this, we first conducted an *in silico* analysis to identify putative master regulators that could potentially bind to the promoter sites of the DEGs. Given the significant and robust changes, our analysis was primarily focused on the unique DEGs observed in the G3D group or those shared with the Green group.

**FIGURE 3:**
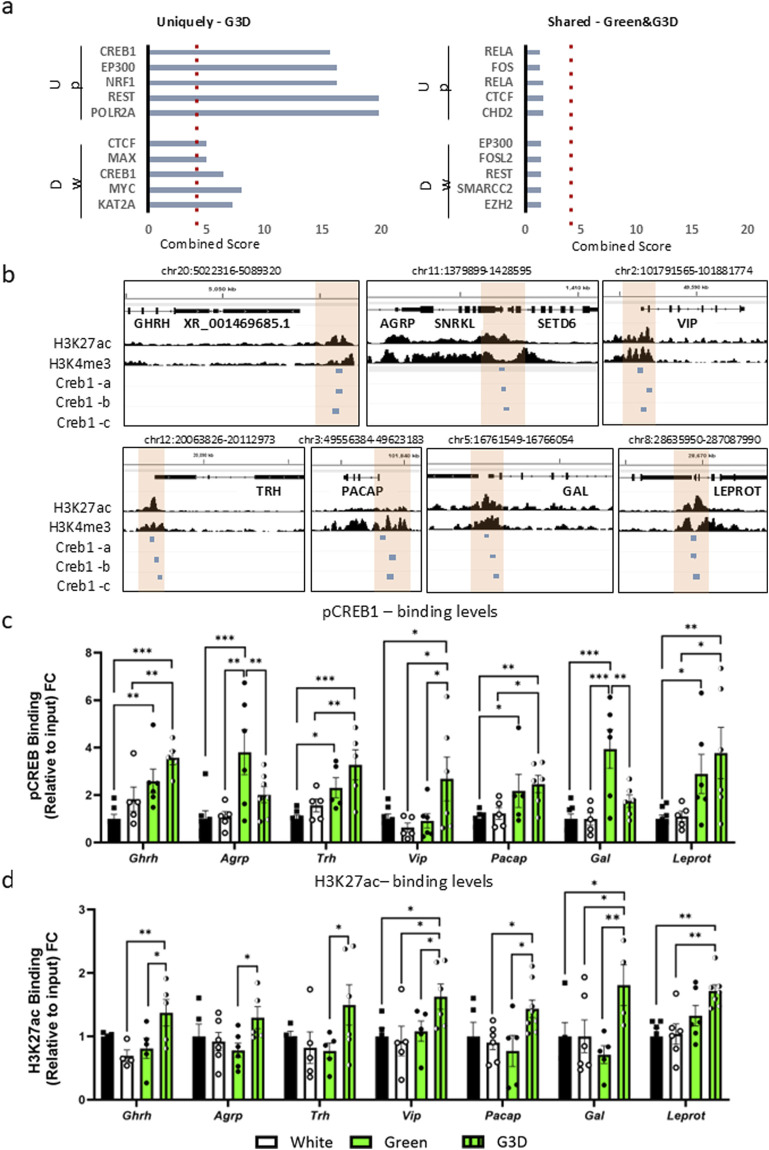
GMI exposure promotes augmented binding of phosphorylated CREB1 (pCREB1) and higher levels of Histone H3 Lysine 27 acetylation (H3K27ac) at gene promoters involved in growth, appetite, and metabolism in the G3D group. **(a)**. In-silico analysis of transcriptional master regulators of uniquely up- or downregulated DEGs identified in the G3D group (left panel) and shared DEGs between the Green and G3D groups (right panel). **(b)**. IGV genome browser tracks presenting genomic loci of the Ghrh, Agrp, Vip, Trh, Pacap, Galanin, and Leprot genes on the Galgal 6.0 genome track. Pan et al. (2023), ChIP-seq tracks from the hypothalamus presenting H3K27ac enrichment, which demarcates active enhancers and promoters, and H3K4me3 peaks, which primarily demarcate active promoters. Promoter regions are indicated by orange rectangles. Three putative CREB1 motif sequences are presented by blue rectangles. Binding levels of **(c)**. pCREB1 and **(d)**. H3K27ac were assessed via ChIP-qPCR with designated primers aligned to the identified promoter/CREB1 sites of Ghrh, Agrp, Vip, Trh, Pacap, Galanin, and Leprot. ChIP enrichment data are presented as Log2FC from the Control group (scatter line). Data are presented as mean ± SEM. Significant effect between groups is indicated by *p < 0.05, **p < 0.01, ***p < 0.001, ****p < 0.00001, using One-way ANOVA test with LSD’s multiple comparisons.

As illustrated in Figure 3a, CREB1, KAT2A, MYC, and EP300 emerged as central modulators among the unique DEGs observed in the G3D group. CREB1 is known for its prominent expression in activated neurons and its interaction with numerous epigenetic modifiers, such as CREB-binding protein (CREBBP; also known as CBP or KAT2A) and E1A-binding protein P300 (EP300; also known as P300 or KAT2B). Both CBP and EP300 harbor histone acetyltransferase (HAT) domain, and their activation results in elevated acetylation levels, predominantly on histone three at lysine 9 and 27 (H3K9ac, H3K27ac) (Altarejos et al., 2011). These epigenetic modifications enhance chromatin accessibility at genomic regulatory sites and stimulate increased transcription of nearby genes. In contrast, no statistically significant motifs were found in the shared DEGs between the Green and G3D groups (Figure 3a, right panel). Next, we curated a list of target DEGs, both shared and unique, that exhibit strong associations with appetite, growth metabolism, and immunity (*Agrp*, *Vip*, *Pacap*, *Gal* and *Laprot*. Figures 1e, 2c,d). Given previous results and despite their lack of significance in our RNA-seq analysis, we also investigated the *Ghrh* and *Trh* genes. Leveraging previously published chromatin immunoprecipitation followed by sequencing (ChIP-seq) data from the chicken hypothalamus (Pan et al., 2023), we first identified accessible promoter regions of the target genes enriched with DNase peaks, H3K27ac and H3K4me3 marks (Supplementary Figure S2, 3b). Further analysis with three different CREB1 motif sequences confirmed potential binding sites in all the aforementioned promoter regions (Figure 3b). An intriguing discovery pertains to the enrichment of CREB1 and H3K27ac marks near the promoter site of Leprot, which exhibited decreased levels in our RNA-seq analysis (Figure 1e).

Next, by utilizing ChIP-qPCR, we measured the enrichment levels of pCREB1 at the identified promoters in all tested groups (i.e., Control, White, Green, and G3D) (Figure 3c). Accordingly, when compared to the dark-incubated control group, we detected a significant elevation in pCREB1 binding levels at the promoter sites of Vip, Leprot, Gal, and Pacap in the G3D group (Figure 3c). Additionally, the Green group also displayed a significant elevation at the promoter sites of Vip, Gal, Pacap, and Ghrh (Figure 3c), in comparison to the controls. However, apart from the Gal and Agrp promoter sites, the increase in pCREB1 binding was less pronounced compared to the elevation observed in the G3D group. An additional immunofluorescence analysis using a CREB1 antibody was conducted on hypothalamic sections from each experimental group. Nuclear CREB1 puncta co-localized with DAPI were quantified as a proxy for p-CREB1, while cytosolic puncta located >3 µm from DAPI were used to assess non-phosphorylated CREB1. This analysis revealed a significant increase in the nuclear-to-cytosolic CREB1 ratio in the G3D group compared to controls (Supplementary Figure S3). These findings are consistent with our ChIP and transcriptomic data, supporting elevated nuclear p-CREB1 levels in response to GMI exposure during late embryogenesis.

While several epigenetic modifications (such as H3K9ac) are generally regarded as relatively stable around gene promoters, H3K27ac is characterized as a more dynamic modification, often associated with environmental stimuli and/or context-dependent neuronal activity (Gao et al., 2020). Thus, in the next phase, we assessed the dynamics of H3K27ac enrichment at the same promoters. Consistent with the elevated pCREB1 binding levels and mRNA expression, we detected a significant increase in H3K27ac levels at the promoter sites of Ghrh [F (3,14) = 3.806, p = 0.0347], Vip [F (3,17) = 3.169, p = 0.0513], Pacap [F (3,20) = 3.066, p = 0.0515], Gal [F (3,16) = 3.365, p = 0.0449] and Leprot [F (3,21) = 6.021, p = 0.004] in the G3D group (G3D vs. Control or White, Figure 3d). Agrp and Trh exhibited a non-significant increase in H3K27ac levels (Agrp: [F (3,18) = 1.936, p = 0.1599], Trh: [F (3,17) = 0.9518, p = 0.4378]). Interestingly, no significant increase in H3K27ac levels was detected at any of the promoter sites in the Green group compared to the controls (Green vs. Control, Figure 3d).

Collectively, our results suggest that GMI promotes increased hypothalamic transcription of GPCRs, calcium-dependent kinases (such as Pacap and Camk2a), and CREB1 coactivator genes (Figures 2c,d). These factors may contribute to elevated phosphorylation of CREB1, which then acts as a scaffold for coactivators like EP300/CBP. As a result, this interaction enhances acetylation at H3K27 within specific gene promoters primarily linked to growth, appetite, and metabolism.

### 3.3 Initial in-ovo GMI exposure enhances hypothalamic sensitivity to green light post-hatching

When established during early developmental stages, epigenetic modifications are often regarded as stable and capable of exerting lasting effects on an organism’s phenotype. Therefore, we examined whether in-ovo exposure to GMI induces persistent changes that enhance hypothalamic responsiveness to green light post-hatching.

**FIGURE 4:**
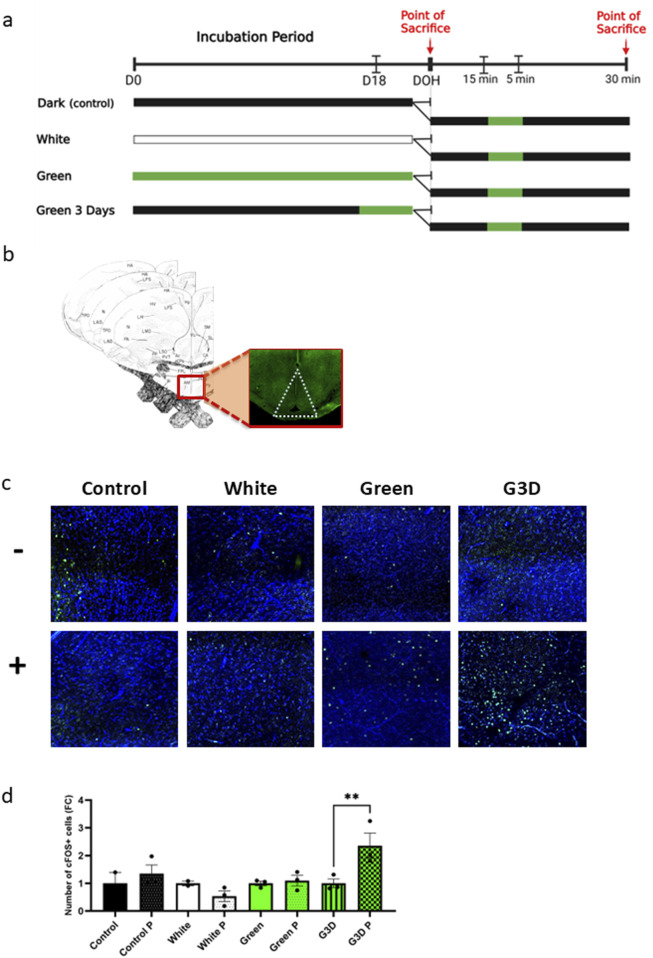
Exposure to GMI in late embryonic stages primes the hypothalamus for post-hatch enhanced sensitivity to GMI **(a)** Illustration of the experimental design; 12 broiler chicks at day of hatch- three from each treatment group (Control, White, Green, and G3D) were subjected to a short pulse of GMI in addition to three other chicks from each treatment group that were sacrificed right after hatch. **(b)** Illustration of the exact location of the area of interest (hypothalamus) in the avian brain for c-FOS positive cells analysis, including the illustrations of the correct coordinates of the hypothalamus-plates A 7.8, A 8.0, and A 8.2 (Kuenzel and Masson, 1988). **(c)** Representative images of immunofluorescence staining of DAPI and c-FOS positive cells (GFP) in the hypothalamus in both chicks that were (+) and were not exposed (−) to a single pulse of GMI from each treatment group. **(d)** Number of c-FOS positive cells in the hypothalamus in both exposed and deprived of a single GMI pulse at DOH (n = 2–3). Data are presented as mean ± SEM. Significant effect between groups is indicated by *p < 0.05, **p < 0.01, ***p < 0.001, ****p < 0.00001, using One-way ANOVA test with LSD’s multiple comparisons.

To test this, 24 broiler eggs were incubated under four experimental conditions (Control, White, Green, G3D, N = 6 per treatment). At hatch, half of the chicks from each treatment group were sacrificed immediately, while the remaining half were exposed to a 5-min pulse of green monochromatic light followed by 30 min of darkness before sacrifice (Figure 4a). Whole brain sections were collected for immunofluorescence staining to assess hypothalamic responsiveness by measuring cFOS levels (Figure 4b), an immediate early gene widely used as a marker of neuronal activity (Bouwknecht et al., 2007).

Remarkably, in response to the green light pulse, the G3D group exhibited a significant increase in the number of cFOS-positive cells compared to their counterparts not exposed to the pulse [F (7,14) = 4.275, p = 0.01], (Figures 4c,d). In contrast, while a mild increase was observed, no significant differences in cFOS expression were detected between the Green and Control groups following the post-hatch green light pulse (Figures 4c,d). These findings suggest that the initial in-ovo exposure to GMI primed the brain’s circuitry for future responses. This priming established a lasting imprint that enhanced the hypothalamus’s sensitivity to subsequent green light pulses. Furthermore, this highlights the critical importance of the final 3 days of incubation as a key developmental window for establishing this enhanced sensitivity.

### 3.4 Preceding blue illumination reduces green opsin levels in the retina and inhibits hypothalamic epigenetic changes induced by GMI

The concept that visual stimuli received by the retina propagate to deeper brain regions such as the hypothalamus or hippocampus has been demonstrated in several studies (Wei et al., 2022; Adaikkan and Tsai, 2020). Thus, we posit that photoreceptors responsible for sensing green light in the retina initiate this process by transmitting information to other brain regions, thereby facilitating hypothalamic adaptation. Consequently, we hypothesize that disrupting the function of green light-sensitive photoreceptors will inhibit the associated epigenetic modifications.

**FIGURE 5:**
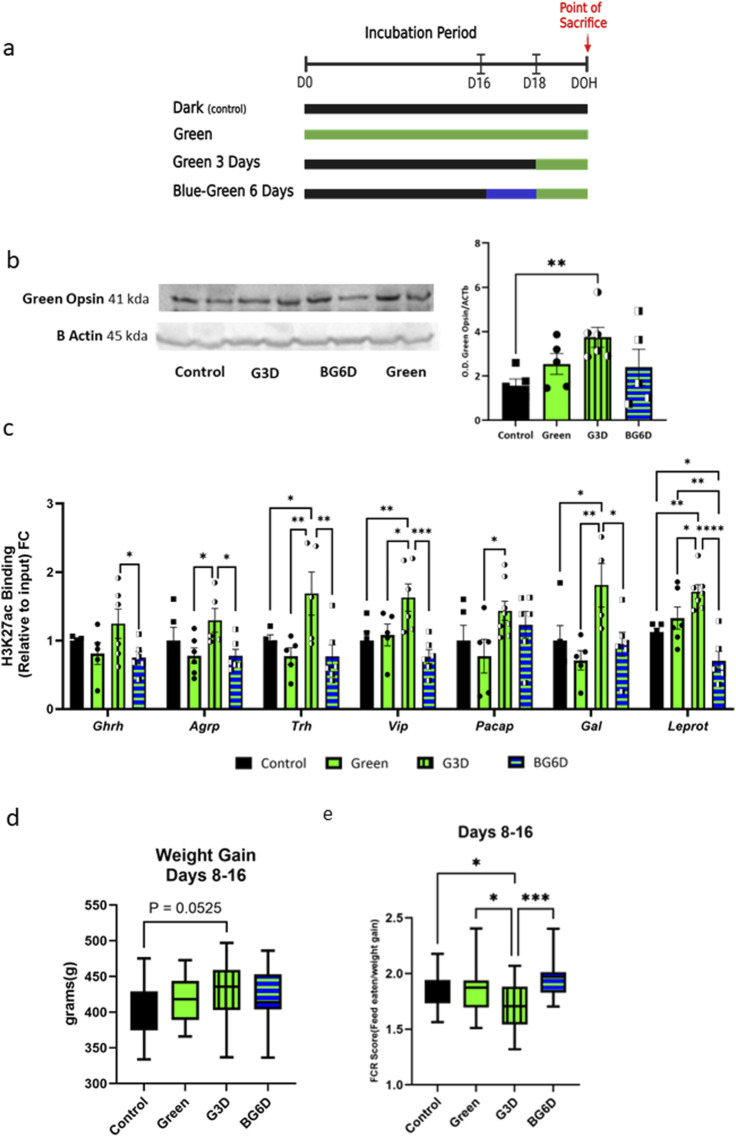
Pre-exposure to blue light, which disrupts green photoreceptor activity, nullified hypothalamic epigenetic modifications in the G3D group **(a)**. Illustration of the experimental design. fertile broiler eggs were divided into four groups (i) dark incubation (Control), (ii) G3D (iii) Green and (iv) blue illumination for 3 days beginning from embryonic day 16 (E16), followed by an additional 3 days of green light exposure (E18-21) (BG6D). **(b)**. Retinal green opsin levels (right eye) of male chicks from all treatment groups, measured via Western blot analysis at DOH (n = 5–6). Data were normalized to β-actin. **(c)**. Binding levels of **(c)**. pCREB1 and **(d)**. H3K27ac were assessed via ChIP-qPCR with designated primers aligned to the identified promoter/CREB1 sites of Ghrh, Agrp, Vip, Trh, Pacap, Galanin, and Leprot. ChIP enrichment data are presented as Log2FC from the Control group (scatter line). **(d)**. The body weight (BW) of each animal was measured at day 8 and day 16 (n = 13–16), and the delta between the two measurements was calculated to examine weight gain in those days. **(e)**. Food conversion ratios (FCRs), a metric reflecting the metabolic utilization of food consumed per unit of body weight were measured in intervals of 6–8-day blocks (n = 11–16). Data are presented as mean ± SEM. Significant effect between groups is indicated by *p < 0.05, **p < 0.01, ***p < 0.001, ****p < 0.00001, using One-way ANOVA test with LSD’s multiple comparisons.

Previous studies in humans, mice, and avian species have demonstrated that prolonged exposure to blue wavelengths can bleach green light-sensitive photoreceptors and impair their function (Grimm et al., 2001; Grimm et al., 2000). Therefore, fertile broiler eggs were randomly assigned to four groups: (i) dark incubation (Control), (ii) G3D, (iii) Green, and (iv) blue illumination for 3 days starting from embryonic day 16 (E16), followed by an additional 3 days (E18-21) of green light exposure (BG6D) (Figure 5a). At the day of hatch (DOH), chicks were sacrificed, and two hemispheres of the hypothalamus, along with the retina from the right eye (Rogers, 1990) were collected for further examination.

Western blot analysis of retinal protein levels showed that acute exposure (G3D) led to an increase in green opsin abundance, while chronic exposure (Green) did not significantly change the overall amount compared to the control group (Figure 5b; Supplementary Figure S4). These observations suggest that continuous exposure to GMI throughout the incubation period may lead to opsin -saturation or -habituation due to overexposure. These outcomes can hinder the full expression of GMI effects, thereby impeding the molecular changes in this group. Notably, the levels of green opsin in the BG6D group were comparable to those in the Green and control groups (Figure 5b), confirming that prior blue illumination affected the amount and presumably the responsiveness of green opsins, thereby hindering the effects of GMI.

Next, we assessed H3K27ac levels at the promoter sites in all experimental groups. Remarkably, prior exposure to blue light before GMI (BG6D) completely abolished the epigenetic effects, as indicated by H3K27ac levels comparable to those in the control group and significantly different from G3D (Ghrh: [F (3,17) = 2.315, p = 0.1123]; Agrp: [F (3,18) = 2.883, p = 0.0644]; Trh: [F (3,16) = 4.791, p = 0.0144]; Vip: [F (3,18) = 6.119, p = 0.0047]; Gal: [F (3,16) = 4.493, p = 0.0181]; Leprot: [F (3,20) = 12.41, p < 0.0001]; Figure 5c). This outcome stands in sharp contrast to the significant increase observed in the G3D group compared to controls (Figure 5c). Collectively, these results suggest that GMI-induced epigenetic changes are at least partially mediated through direct or indirect neuronal pathways linking the retina to the hypothalamus.

### 3.5 Phenotypic effects of GMI and the impact of preceding blue illumination (BG6D)

In the final step, we assessed whether GMI influences phenotype and whether blue illumination prior to green illumination (BG6D) would negate these effects. During the first and second weeks post-hatching (days 8–16), we measured changes in body weight (BW) gain and food conversion ratio (FCR), an index of metabolic efficiency representing the amount of food consumed relative to body weight gain.

Our findings revealed a near-significant increase in BW delta in the G3D group compared to controls (F (3,53) = 1.334, p = 0.2730, Figure 5d). However, no significant differences in BW were observed in the Green or BG6D groups (Figure 5d). Additionally, the G3D group exhibited a significantly lower FCR during the days 8–16 period compared to all other groups (F (3,56) = 4.895, p = 0.0043). Consistent with our molecular findings, the BG6D group did not display significant differences in FCR in comparison to the other groups.

These results corroborate our transcriptomic and epigenetic data, indicating that in-ovo exposure to GMI enhances energy utilization and metabolism, particularly during early post-hatch development stages.

## 4 Discussion

Over the years, farmed birds have been selectively bred for performance traits, primarily through genetic selection. This approach has led to substantial increases in both body weight and growth rates. However, to sustain such rapid growth, food consumption has risen significantly. As feed costs account for 60%–70% of production expenses in the poultry industry, there is a continuous drive to enhance growth efficiency and optimize feed-to-energy conversion in broilers. At the same time, efforts focus on improving overall health resilience and ensuring the welfare of the animals. Our study demonstrates that GMI exposure during critical developmental windows induces hypothalamic epigenetic and transcriptional alterations that augment phenotypic plasticity of growth and metabolic efficiency. We demonstrated that these effects are at least partially mediated through the retinal green-opsin signaling and highlighting the importance of precise temporal and spectral light exposure in modulating developmental trajectories.

These findings are supported by several key observations. First, our RNA-seq analysis at DOH revealed minimal transcriptional changes in the White group compared to the controls. Moreover, although we found only 34 overlapping DEGs between the GMI–treated groups (Figures 1c, 2a), the Green group showed the same pattern of increase and decrease in gene expression as the G3D group, but most of these changes did not reach statistical significance (Figure 1b). Accordingly, many of these DEG’s are directly involved in the regulation of the HPS axis (such as Pacap, Sst, and Vip, Leprot and Gal), appetite and metabolism (Agrp, Igfbp5).

Alongside these findings, we observed an increase in the expression of Nfkbib a key regulator known for its role in suppressing inflammation (Huang et al., 2020). This aligns with previous studies demonstrating that exposure to artificial green illumination is associated with reduced hypothalamic inflammation and enhanced growth and bone quality in laying hens (Wei et al., 2022). Collectively, these findings not only validate our previous results (Dis et al., 2021) but also suggest broader-scale hypothalamic alterations induced by GMI, which depend upon the developmental phase and duration of this manipulation.

GMI adaptations included heightened transcriptional activation of hypothalamic G-protein mediated events and calcium-kinase dependent pathways, such as Camk2a and Pacap (Figure 2d). Previous research has extensively demonstrated that CaMKII-mediated CREB phosphorylation is crucial for facilitating molecular memory in neurons (Kandel, 2012). As a transcription factor, CREB recruits coactivators, initiating an epigenetic modification phase that increases chromatin accessibility, thereby enhancing transcriptional efficiency.

To investigate the direct involvement of these factors, we examined their roles across different groups. Accordingly, animals exposed to GMI exhibited a significant increase in both pCREB1 binding and H3K27ac levels at the promoters of genes associated with growth, appetite, and metabolism (Figure 3). These findings underscore the specificity of GMI while also highlighting the importance of exposure timing and duration, as evidenced by the pronounced molecular, epigenetic, and transcriptional profiles in the G3D animals.

Next, we investigated whether hypothalamic responsiveness to GMI was enhanced following epigenetic reprogramming. For that, we used cFOS immunostaining as a widely recognized marker of neuronal activation. Our results showed that while no significant changes in cFOS expression were observed in the Green or Control groups, the G3D group displayed a notable increase in both the number and intensity of cFOS-positive cells following a post-hatch green light stimulus (Figures 4c,d). These results suggest that the initial in-ovo exposure to GMI established a lasting imprint that enhanced the hypothalamus’s sensitivity to subsequent green light pulses. Thus, similar to how an initial environmental cue can prime the brain’s circuitry for future responses, early exposure to green illumination in avian embryos appears to prime the hypothalamus for increased neuronal activation in response to later green light stimuli.

Given the obtained results, we were specifically interested in elucidating the biological factors that distinguish between the Green and the G3D groups. The concept that visual stimuli received by the retina propagate to deeper brain regions such as the hypothalamus or hippocampus has been demonstrated in several previous studies (Rogers, 1990; Madison et al., 2024). Thus, we posit that photoreceptors responsible for sensing green light in the retina initially mediate this process by transmitting information to other brain regions, thereby facilitating ‘molecular adaption’ in the hypothalamus. In alignment, acute exposure (G3D) led to an increase in the abundance of retinal green opsins, while chronic exposure (Green) did not significantly alter the overall amount compared to the control group (Figure 5B). Opsins in the retina can undergo inactivation following increased or prolonged exposure to light of different wavelengths (Grimm et al., 2001). Therefore, chronic exposure to GMI may significantly reduce the number of functional green photoreceptors (Figure 5B). Moreover, in line with the levels of retinal green opsins, hypothalamic H3K27ac levels were also comparable to the control group, contrasting with the observed increase in the G3D group.

Additionally, considering that blue illumination can bleach retinal green opsins and disrupt their function (Grimm et al., 2001; Grimm et al., 2000), we designed another experiment where embryos were first exposed to blue light for 3 days, followed by 3 days of sequential exposure to GMI (BG6D). The levels of retinal green opsins in the BG6D group were comparable to those in the Green and control groups (Figure 5b), confirming that prior blue illumination affected the number and likely the responsiveness of green opsins. Moreover, this manipulation impeded the effects of GMI, as hypothalamic H3K27ac levels remained comparable to those of the control group. Collectively, this suggests that green opsin-mediated signaling plays a crucial role in GMI-induced epigenetic and transcriptional alterations. Furthermore, this observation implies that the process is sensitive to duration and disturbances; increased exposure or inactivation through blue illuminations can hinder the full expression of GMI effects.

Last, phenotypically, the G3D group exhibited a near-significant increase in BW gain, with corresponding improvements in food conversion ratio (FCR), indicating enhanced energy utilization and metabolism. Notably, the BG6D group, exposed to blue light before GMI, showed no significant changes in BW, corroborating the disruption of GMI effects through prior blue illumination. Therefore, further research will focus on exploring how these changes can be stabilized and their effects prolonged.

## 5 Conclusion

Our study offers new insights into how specific wavelengths and precise timing of light exposure during critical developmental windows can translate into broader systemic effects, inducing epigenetic and transcriptional changes that enhance phenotypic plasticity. From a broader perspective, these findings illuminate key aspects of developmental biology and highlight the crucial role of epigenetics in shaping organismal responses to their environment.

## Data Availability

All data generated or analyzed during this study are included in the article/supplementary material. In addition, RNA sequencing data generated in this study are available at Gene Expression Omnibus (GEO) under accession number GSE273328.

## Glossary

ADAM10: metallopeptidase domain 10
AGRP: Agouti-related protein
AMPK: AMP-activated protein kinase
APMAP: Adipocyte Plasma Membrane Protein
BG6D: Blue green 6 days
BW: Body weight
CAMK2a: Calcium/Calmodulin Dependent Protein Kinase II Alpha
ChIP-SEQ: Chromatin immunoprecipitation-sequencing
CREBBP: CREB-binding protein
DEG: Differentially expressed gene
DOH: Day of hatch
E16: Embryonic day 16
E18: Embryonic day 18
EP300: E1A Binding Protein P300
ERPR: Extra-retinal photoreceptors
FCR: Food conversion ratio
G3D: Green 3 Days
GAL: Galanin
GH: Growth hormone
GHR: Growth hormone receptor
GHRH: Growth hormone releasing hormone
GMI: Green monochromatic illumination
GO: Gene Ontology
GPCR: G protein-coupled receptors
H3K27ac: Histone H3 Lysine 27 acetylation
H3K4me3: Histone H3 Lysine 4 methylation
H3K9ac: Histone H3 Lysine 9 acetylation
HAT: Histone acetyltransferase
HPCAL1: Hippocalcin-like protein 1
IGF1: Insulin-like growth factor 1
IGF2R: Insulin-like growth factor 2 receptor
IGFBP5: Insulin-like growth factor-binding protein 5
LAMP2: Lysosome-associated membrane protein 2
LEPR: Leptin receptor
LEPROT: Leptin Receptor Overlapping Transcript
NFKBIB: NF-kappa-B inhibitor beta
PACAP: Pituitary adenylate cyclase-activating polypeptide
PCA: Principal component analysis
pCREB1: phosphorylated CREB1
SST: Somatostatin
TRH: Thyrotropin-Releasing Hormone
VIP: Vasoactive Intestinal Peptide
