# Supplementary figures and images for: Light-induced epigenetic modifications in the hypothalamus during avian embryonic development enhance phenotypic plasticity
Source: Front Cell Dev Biol. 2025 Jun 26;13:1573705. doi: 10.3389/fcell.2025.1573705 (PMC12241980; doi:10.3389/fcell.2025.1573705)

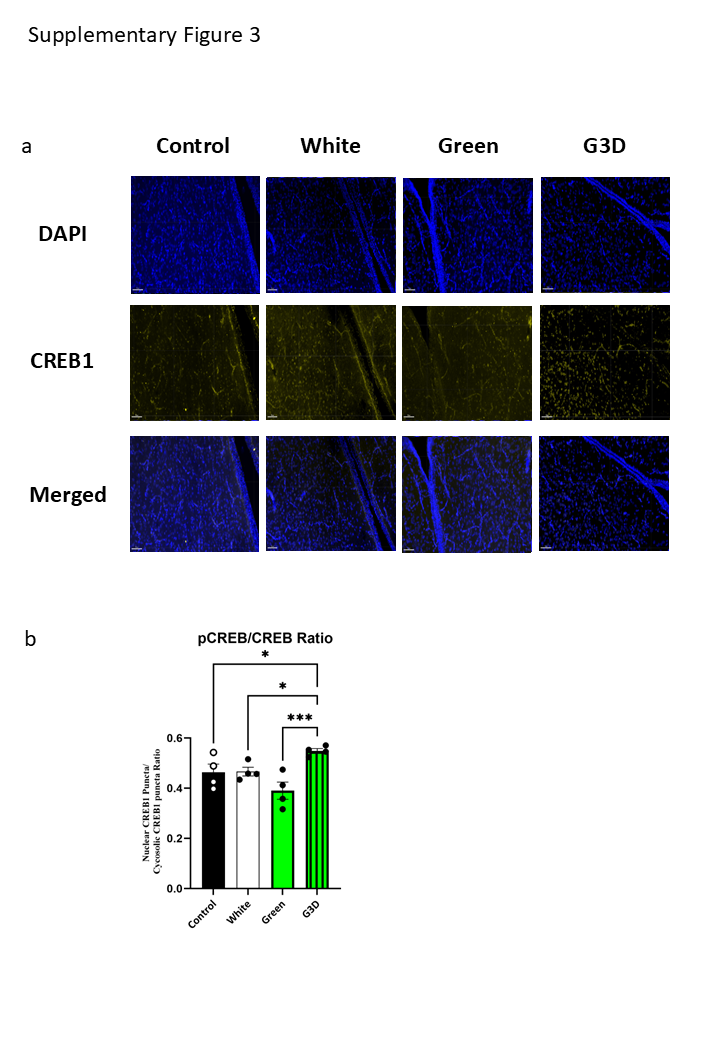

Supplement: Supplementary file 1 [file Image3.tif]

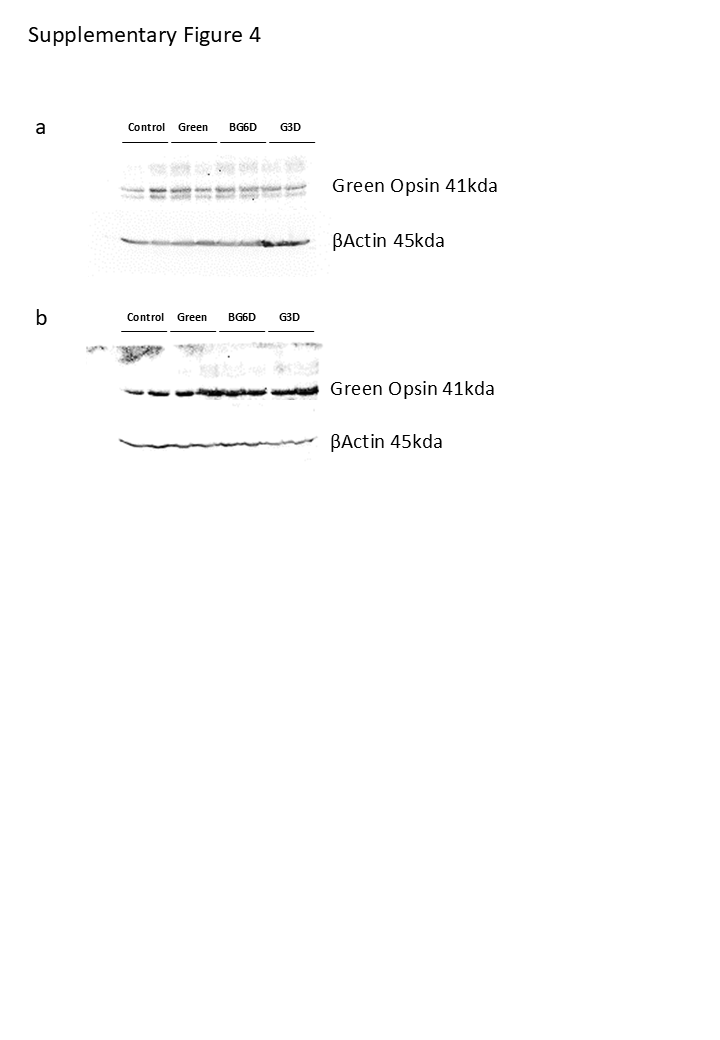

Supplement: Supplementary file 2 [file Image4.tif]

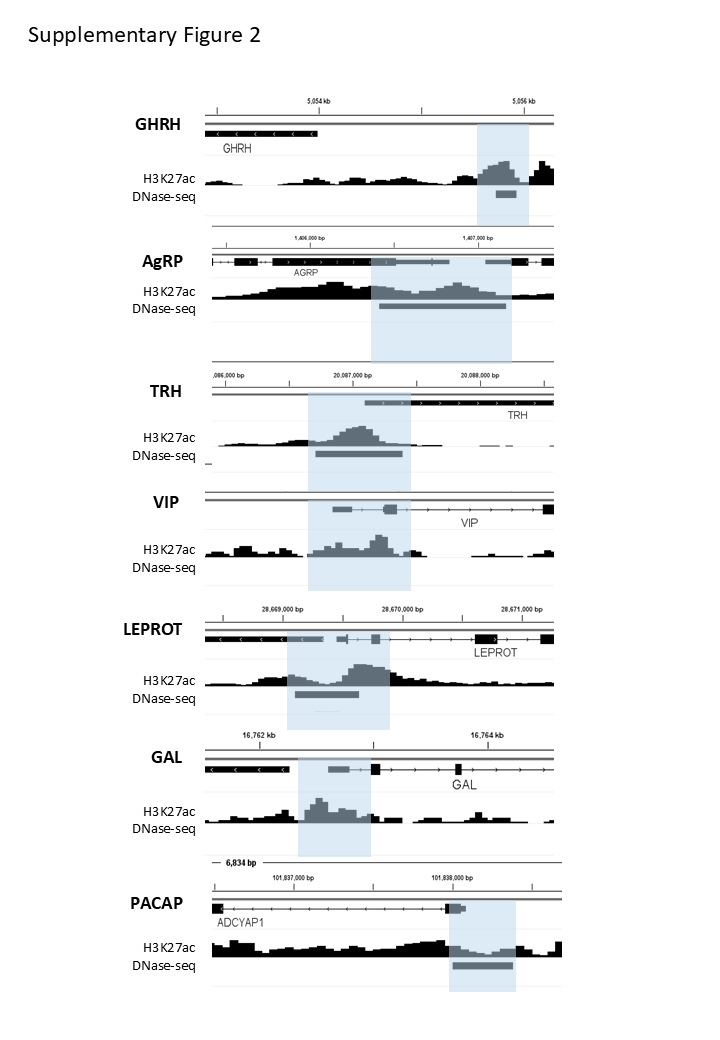

Supplement: Supplementary file 3 [file Image2.tif]

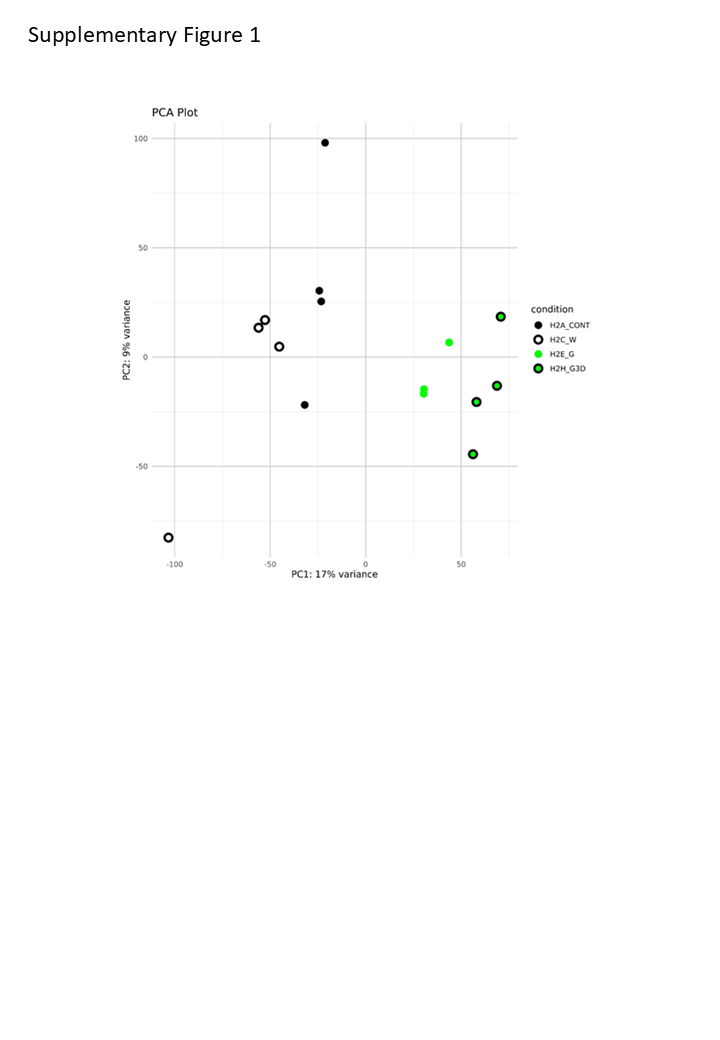

Supplement: Supplementary file 4 [file Image1.tif]
